# Discrimination of *Baphicacanthis* *Cusiae* Rhizoma et radix and its adulterant species and establishment of an assay method for quality control

**DOI:** 10.1186/s13020-023-00777-x

**Published:** 2023-06-02

**Authors:** Yue Li, Qiong-Xi Yu, Lee-Fong Yau, Guo-Kai Huang, Jing-Guang Lu, Xiao-Xiao Liu, Zhi-Hong Jiang, Jing-Rong Wang

**Affiliations:** 1 State Key Laboratory of Quality Research in Chinese Medicines, Macau Institute for Applied Research in Medicine and Health, Macau University of Science and Technology, Taipa, 999078 Macao China; 2 State Key Laboratory of Dampness, Syndrome of Chinese Medicine, The Second Affiliated Hospital of Guangzhou University of Chinese Medicine, Guangzhou, 510000 China; 3 Guangdong-Hong Kong-Macau Joint Lab On Chinese Medicine and Immune Disease Research, Guangzhou, 510000 China; 4 Guangdong Institute for Drug Control, Guangzhou, 510663 China

**Keywords:** UHPLC Q-TOF-MS, Chromatographic fingerprint, Quantitative markers, *Isatis indigotica* Fort., 2-Benzoxazolinone, Acteoside

## Abstract

**Background:**

*Baphicacanthis* *Cusiae* Rhizoma et Radix, commonly known as Nan-Ban-Lan-Gen (NBLG), is an essential traditional Chinese medicine that possesses diverse bioactivities, particularly noteworthy for its antiviral properties. Although NBLG has been listed in the Chinese Pharmacopoeia as an independent Chinese medicine, the establishment of a comprehensive quality standard for NBLG remains elusive. The absence of assay for marker compound in its quality standards has led to the lack of corresponding quality control measures for NBLG-containing preparations, and its discrimination from adulterant species in the market which thereby can significantly impact the efficacy and safety of NBLG-containing products.

**Methods:**

Ultra-high performance liquid chromatography (UHPLC) coupled with quadrupole-time-of-flight mass spectrometry (Q-TOF-MS) was employed for comprehensive profiling of the chemical constituents of NBLG, the stem of *Baphicacanthus cusia* (Nees) Bremek (NBLJ), and the roots of *Isatis indigotica* Fort. (Bei-Ban-Lan-Gen, BBLG). Additionally, multivariate analysis was conducted to compare the chemical components of NBLG with those of NBLJ and BBLG. Furthermore, we established an optimized and validated HPLC method to obtain the fingerprint of NBLG and quantify the content of 2-benzoxazolinone and acteoside in the samples.

**Results:**

A total of 73 compounds belonging to six classes were assigned in NBLG, with alkaloids being the most abundant and diverse species. High compositional similarities with significant differences in content were observed between NBLG and NBLJ. Moreover, the chemical profile of BBLG markedly differed from that of NBLG. An informative high performance liquid chromatography (HPLC) fingerprint of NBLG comprising seven characteristic peaks that can be used for quality assessment was established. Notably, we propose a quality control standard for NBLG, stipulating that the limit of content in dry weight for both 2-benzoxazolinone and acteoside should not be less than 0.010%.

**Conclusion:**

This study provides the most comprehensive chemical information to date on NBLG, offering valuable insights into its authentication and quality control. Our findings highlight the importance of comprehensive chemical profiling to differentiate potential substitutions and adulterations of herbal medicines, particularly when the original source is scarce or unavailable. These results can aid in the development of quality control measures for NBLG-containing preparations, ensuring their safety and efficacy.

**Supplementary Information:**

The online version contains supplementary material available at 10.1186/s13020-023-00777-x.

## Background

Baphicacanthis Cusiae Rhizoma et Radix, commonly known as Nan-Ban-Lan-Gen (NBLG), is a traditional Chinese medicine derived from the root and rhizome of *Baphicacanthus cusia* (Nees) Bremek (*B. cusia*). NBLG exhibits a bitter taste and cold nature, and has traditionally been used to treat sore throat and fever due to its promising antiviral, anti-inflammatory, and antibacterial properties [1].

NBLG was initially included in the Chinese Pharmacopoeia (CHP) as one of the multiple resources of Ban-Lan-Gen (BLG), together with the roots of *Isatis indigotica* Fort. (Isatidis Radix, Bei-Ban-Lan-Gen, BBLG). Both of these medicinal materials have been traditionally used for clearing heat and resolving toxin. However, evidence from studies on their plant sources, origins, as well as chemical and pharmacological properties, has revealed notable differences between NBLG and BBLG [2, 3]. For instance, NBLG contains a higher level of indirubin, a bioactive compound with potent anticancer properties, compared to BBLG [4]. As such, NBLG was listed as an independent herbal medicine in CHP since 1995.

NBLG has attracted great research interest because of its notable antiviral activities [5]. Studies have demonstrated its significant inhibitory effect on hemagglutination of influenza virus, while BBLG has almost no obvious inhibition activity [6]. Moreover, the water-soluble components of NBLG possess strong antimicrobial, antiviral, and antiendotoxin properties [7]. During the severe acute respiratory syndrome (SARS) epidemic, NBLG was used as one of the eight antiviral Chinese herbal medicines for the prevention and treatment of SARS [8], highlighting its promising antiviral activity [9]. NBLG is included as a major ingredient in a series of Chinese patent medicines manufactured in China every year. According to information from the National Medical Products Administration and Traditional Chinese Medicine Preparations issued by the Ministry of Health of the People’s Republic of China, ten complex Chinese medicines containing NBLG have been approved for production. More than two hundreds companies have been approved to manufacture these preparations, among which 120 companies use NBLG as the major ingredient for manufacturing anti-cold Chinese medicines [10]. This suggests a large demand for NBLG. However, although NBLG has been independently included in the CHP, its quality standard is not well-established yet. Till now, an assay for the determination of marker compounds is still absent in the quality standard of NBLG in CHP, leading to the lack of corresponding quality control for related Chinese medicine preparations.

Additionally, as NBLG is primarily collected from the wild, its resources are relatively scarce [11], leading to the collection of non-official medicinal parts such as the above-ground stem of *B. cusia* (NBLJ) together with the rhizome and root for usage [12]. In some cases, NBLJ is even used instead of NBLG in the market [13]. In southern provinces of China, BBLG has also been illegally used as a substitute for NBLG in manufacturing [14]. These issues raise concerns regarding the potential efficacy and safety problems of NBLG and NBLG-containing preparations, emphasizing the urgent need for specific quality control measures for NBLG.

In this study, we conducted a comprehensive profiling of the chemical constituents in NBLG, NBLJ, and BBLG using an optimized sample preparation procedure and ultra-high performance liquid chromatography (UHPLC) coupled with quadrupole time-of-flight mass spectrometry (Q-TOF–MS) method. The objective was to discriminate between NBLG and its adulterant species, namely NBLJ and BBLG, by providing evidence of their compositional differences through multivariate analysis. Furthermore, we established a high performance liquid chromatography (HPLC) fingerprint of NBLG using eleven batches of NBLG. Subsequently, we selected ideal chemical markers and quantified them using our optimized and validated HPLC method. As a result, we propose a new quality control standard for NBLG that provides a crucial benchmark for assessing its quality.

## Materials and methods

### Materials and reagents

The plant materials were taxonomically identified and provided by experts from the production area and the Guangdong Institute for Drug Control (GDIDC). Eleven batches of the whole plant of NBLG were primarily collected from Guangdong, with additional collections from Guangxi, Yunnan, and Sichuan. The root and rhizome, as well as the stems, were separated from each individual plant to obtain NBLG and NBLJ, respectively. Furthermore, ten batches of BBLG were obtained from Anhui, with additional collections from Henan, Hebei, Shanxi, and Guangxi. The detailed information related to the plant materials is presented in Table 1.

**Table 1:** Information of eleven batches of *Baphicacanthus cusia* (Nees) Bremek and ten batches of *Isatis indigotica* Fort

Species	Part	Batch no	Origin of sample
*Baphicacanthus cusia* (Nees) Bremek	Root and rhizome (Nan-Ban-Lan-Gen, NBLG)	NBLG-1	Guangdong, China
		NBLG-2	Yunnan, China
		NBLG-3	Guangxi, China
		NBLG-4	Yunnan, China
		NBLG-5	Guangdong, China
		NBLG-6	Sichuan, China
		NBLG-7	Guangdong, China
		NBLG-8	Guangdong, China
		NBLG-9	Guangdong, China
		NBLG-10	Guangdong, China
		NBLG-11	Guangdong, China
	Stem (Nan-Ban-Lan-Jing, NBLJ)	NBLJ-1	Same as NBLG-1
		NBLJ-2	Same as NBLG-2
		NBLJ-3	Same as NBLG-3
		NBLJ-4	Same as NBLG-4
		NBLJ-5	Same as NBLG-5
		NBLJ-6	Same as NBLG-6
		NBLJ-7	Same as NBLG-7
		NBLJ-8	Same as NBLG-8
		NBLJ-9	Same as NBLG-9
		NBLJ-10	Same as NBLG-10
		NBLJ-11	Same as NBLG-11
*Isatis indigotica* Fort	Root (Bei-Ban-Lan-Gen, BBLG)	BBLG-1	Henan, China
		BBLG-2	Henan, China
		BBLG-3	Anhui, China
		BBLG-4	Anhui, China
		BBLG-5	Anhui, China
		BBLG-6	Anhui, China
		BBLG-7	Anhui, China
		BBLG-8	Hebei, China
		BBLG-9	Guangxi, China
		BBLG-10	Shanxi, China

The standards for adenosine, acteoside, 2-benzoxazolinone, indigo, indirubin, 2,4-(1*H*,3*H*)-quinazolinedione, tryptanthrine, and 1*H*-indole-3-carbaldehyde (purity ≥ 98%) were purchased from Shanghai Yuanye Biotechnology Co., Ltd. (Shanghai, China) and Chengdu Must Bio-technology Co., Ltd. (Chengdu, China). HPLC grade methanol (MeOH), acetonitrile (ACN), formic acid (FA), and acetic acid (AA) were obtained from Merck (Darmstadt, Germany). Purified distilled water was obtained using a Milli-Q system (Millipore, Inc., MA, USA).

### Preparation of standard and sample solutions

The stock solutions of the eight standards were prepared in *N,N*-dimethylformamide. They were then further diluted to a concentration of 50 µg/mL for qualitative analysis. To determine the content via HPLC, 2-benzoxazolinone and acteoside were precisely weighted, and a mixed solution containing 50 µg/mL of 2-benzoxazolinone and 100 µg/mL of acteoside was dissolved in *N,N*-dimethylformamide.

For the qualitative analysis, 100 mg of powdered sample was weighed into a PTFE-stopped tube and sonicated with 10 mL of MeOH for 45 min. The mixture was then centrifuged, and the supernatant was collected. For quantitative analysis, 1 g of the powdered sample was accurately weighed into a 50 mL round-bottom flask. Then, 50 mL of MeOH was added, and the mixture was refluxed in a water bath for 60 min. The extracted solution was filtered, and the filtrate was collected for later use. The residue was extracted one more time, and the filtrate was combined with the first extract. The combined extract was evaporated to dryness, and the residue was dissolved in *N*,*N*-dimethylformamide. The volume of the solution was adjusted to 5 mL, and the obtained solution was filtered to yield a sample solution for testing.

The water content in NBLG was measured using the oven-dried method according to the guidelines in the CHP. Briefly, 2–5 g of the powdered sample was put into a flat weighing bottle and accurately weighed. The sample was dried in an oven at 100–105 °C for 5 h with the bottle cap open. Afterward, the bottle was promptly closed and allowed to cool in a desiccator for 30 min. Then, the sample was weighed accurately and dried again under similar conditions for 1 h, followed by successive cooling and weighing. The operation was repeated until the difference between two successive weights did not exceed 5 mg. Finally, the percentage of water content in NBLG sample was calculated based on the weight lost.

### UHPC-Q-TOF–MS conditions for chemicals profiling

Chemical profiling was performed using an Agilent UHPLC-MS system (Agilent Technologies 6545 LC/Q-TOF) equipped with an ESI source. The instrument was operated using Agilent MassHunter Qualitative Analysis B.06.00 software (Agilent Technologies, Santa Clara, CA, USA) for data acquisition and analysis. Chromatographic separation was achieved using an Agilent ZORBAX Eclipse Plus C_18_ column (Rapid Resolution HD, 2.1 × 150 mm, 1.8 µm). The mobile phase comprised of 0.1% FA in water (A) and 0.1% FA in ACN (B), with a flow rate of 0.35 mL/min. The gradient employed was as follows: 0 − 15 min, 2 − 30% B; 15 − 25 min, 30 − 100% B; 25 − 30 min, 100%. The column temperature was maintained at 35 °C, and each sample was injected at a volume of 3 μL. The mass spectrometer was operated in the positive mode with a scanning range of *m/z* 100 − 1700 for both MS and MS/MS analysis. The following settings were used for MS analysis: drying gas temperature of 325 °C; drying gas flow of 10 L/min; nebulizer pressure of 40 psi; sheath gas temperature of 350 °C; sheath gas flow of 11 L/min; fragmentor voltage of 175 V; skimmer voltage of 65 V: octopole RF voltage of 750 V; VCap voltage of 3500 V; and nozzle voltage of 500 V. The targeted MS/MS collision energy was set to 20 eV.

### HPLC conditions for fingerprint analysis and quantitative determination

HPLC was performed using an Agilent 1200 series system. Chromatographic separation was carried out on a Kromasil C_18_ column (250 mm × 4.6 mm, 5 µm). The mobile phase consisted of 0.1% AA in water (A) and ACN (B), with a flow rate of 1 mL/min. Gradient elution was performed using the following conditions: 0 − 30 min, 5 − 20% B; 30 − 35 min, 20% B; 35 − 55 min, 20 − 40% B; 55 − 75 min, 40 − 85% B; 75 − 80 min, 85% B; 80 − 90 min, 85 − 100% B. The column temperature was maintained at 35 ℃, and the injection volume for each sample was 10 μL. UV detection wavelength of 280 nm was used for NBLG HPLC fingerprint analysis, while wavelengths of 274 nm and 332 nm were employed for the quantitative analysis of 2-benzoxazolinone and acteoside, respectively. The Similarity Evaluation System for Chromatographic Fingerprint of Traditional Chinese Medicine (Version 2004A) software was utilized to establish the fingerprint and reference chromatogram for eleven batches of NBLG.

### Method validation for HPLC fingerprint analysis

The method validation for HPLC fingerprint analysis was performed, including precision, reproducibility, and stability experiments. Pooled samples of multiple batches of NBLG served as a quality control (QC) solution. Precision was assessed by determining the QC solution six times in succession. Reproducibility was evaluated by preparing six test solutions from the same batch sample. Stability was determined by analyzing the QC solution with storage at room temperature for 0, 2, 4, 6, 8, 10, 24, 48, and 72 h.

### Method validation for HPLC quantitative analysis

The quantitative analysis method was validated for linearity, limit of detection (LOD), limit of quantitation (LOQ), precision, repeatability, stability, and recovery. Linearity determination involved injecting a series of working solutions into the HPLC system, and the calibration curves of 2-benzoxazolinone and acteoside were generated by plotting peak area (*y*) versus concentration (*x*). The correlation coefficient (R^2^) was calculated. The LODs and LOQs were determined by diluting the working solutions until the value of signal-to-noise ratio (S/N) reaches 3 and 10, respectively. Precision was evaluated by analyzing six replicate injections of a QC sample solution on a single day, while repeatability was assessed by preparing six identical samples in parallel. Stability was assessed by analyzing the same QC sample after preparation for 0, 2, 4, 6, 24, 48, and 72 h. The index for precision, repeatability, and stability evaluation was the relative standard deviations (RSD) of the peak area. To assess the recovery, sextuplicate samples with predetermined amount of 2-benzoxazolinone (212.16 ~ 221.08 μg) and acteoside (387.38 ~ 403.68 μg) were spiked with a mixed standard solution containing 230 μg 2-benzoxazolinone and 337 μg acteoside. After extraction and analysis using the aforementioned method, the percentage recovery was calculated as (amount found – original amount) / amount spiked × 100%.

### Data processing and statistics

The SIMCA 17 software (Sartorius, Germany) was utilized to conduct multivariate statistical analysis. Orthogonal partial least squares discriminate analysis (OPLS-DA) was employed to explore the differences between NBLG and NBLJ, as well as NBLG and BBLG. A reliable model was indicated by cumulative values of R^2^Y and Q^2^Y close to 1, and constituents with variable importance in projection (VIP) > 1 were identified as potential markers for differentiation. All statistical analyses were performed using GraphPad Prism 9 software (GraphPad Software, CA, USA). Comparisons were carried out using a *t*-test, and a two-sided *P*-value of < 0.05 was considered statistically significant.

## Results and discussion

### Optimization of sample preparation

To enhance the efficiency of extraction, a rigorous optimization of the extraction conditions was conducted. Initially, a range of extraction solvents, including chloroform, ethyl acetate, 30%, 50%, 70%, and 100% MeOH, as well as water, were evaluated by assessing the quantity and abundance of peaks in the total ion chromatogram (TIC) associated with each solvent. As a result, 100% MeOH was found to be the most suitable extraction solvent due to its superior extraction efficiency. Subsequently, various extraction methods (ultrasonic extraction, reflux extraction, and Soxhlet extraction), extraction durations (30, 60, and 90 min), and the number of extractions were examined to determine the optimal conditions for achieving maximum extraction efficiency. As a result, the optimal parameters for obtaining sample solutions with enhance extraction efficiency are presented in the section titled “Preparation of standard and sample solutions”.

### Establishment of chemical compositions database for NBLG

To achieve a thorough identification of chemical components within NBLG using MS data, it is imperative to establish a comprehensive database that contains all possible compounds. In this study, we constructed a personal database comprising 217 compounds for NBLG identification by referring to the components of the Acanthaceae family listed in the Dictionary of Natural Products (Chapman & Hall/CRC Press, Boca Raton, FL, USA) and conducting a systematic literature review of NBLG and BBLG [3, 15–17].

### Structural interpretation of constituents based on high-resolution MS and MS/MS data

**Fig. 1:**
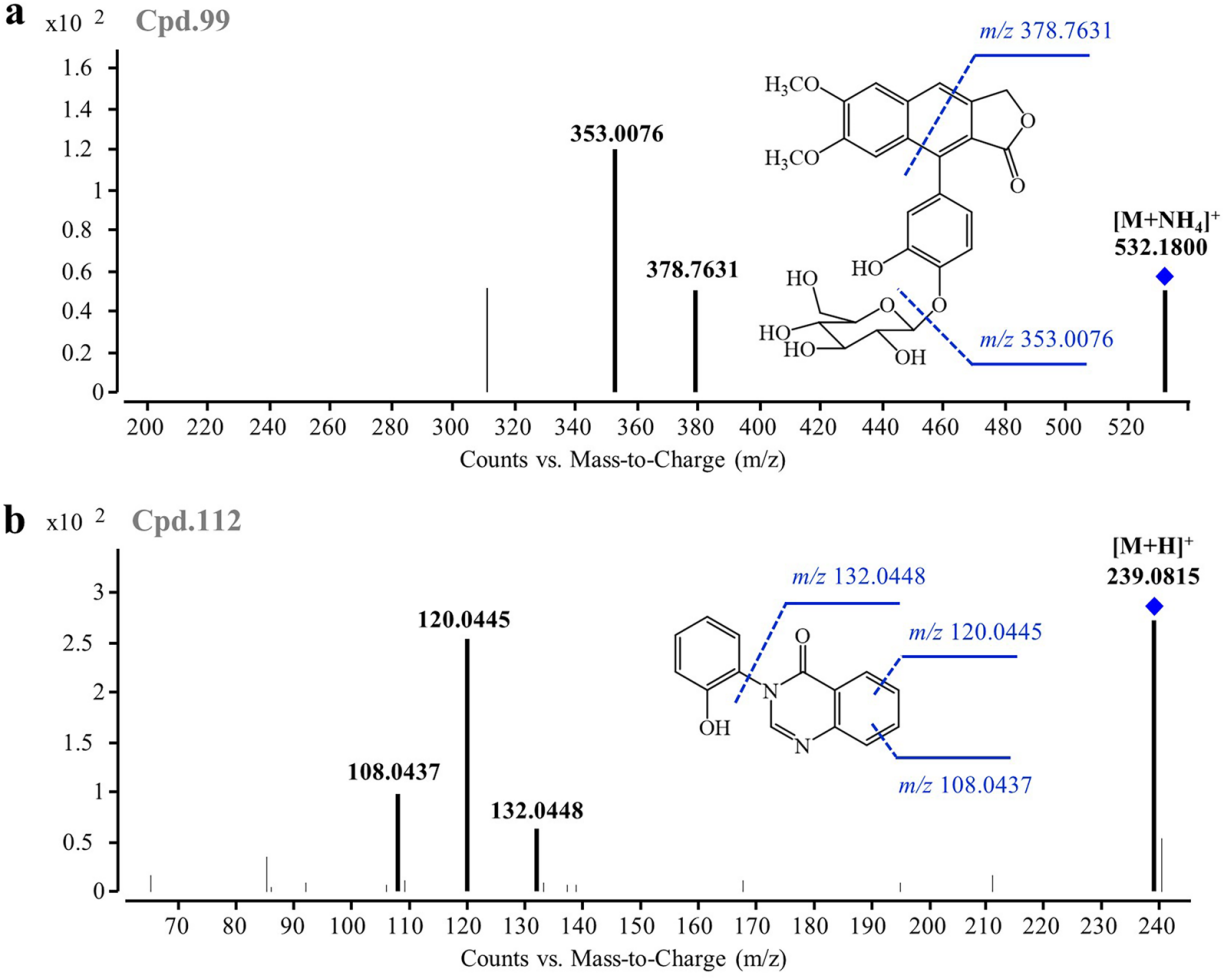
MS/MS spectra and fragmentation pathway of two representative constituents. **a** Cpd.**99** (procumbenoside L) and **b** Cpd.**112** (3-(2-Hydroxyphenyl)-4(3*H*)-quinazolinone)

The “Find by Formula” algorithm in Agilent MassHunter software was employed to comprehensively profile the chemical composition of NBLG. The positive ESI mode was primarily utilized for compound analysis, given that most compounds exhibited higher responses in the positive mode compared to the negative mode. For each compound, the matched score was calculated based on its accurate mass (typically [M + H]^+^, [M + Na]^+^ and [M + NH_4_]^+^ under the positive scan mode of UHPLC Q-TOF–MS, using theoretical values obtained from our personal database), isotopic abundance, and isotopic spacing. The matched compounds were further characterized based on their MS/MS data, resulting in the tentative characterization of a total of 73 compounds in NBLG. The extracted compound chromatogram (ECC) of the QC sample of NBLG is presented in Fig. 3b, and the MS and MS/MS data of the identified components are summarized in Additional file 1: Table S1. Among the identified compounds, 36 were reported for the first time in NBLG (marked with “#” in Additional file 1: Table S1). The identification of a representative compound from the lignan and alkaloid is shown as follows:

Cpd.**99** (t_R_ = 13.45 min) was identified based on an accurate [M + NH_4_]^+^ ion at *m/z* 532.1800 (-2.43 ppm). Its molecular formula was determined as C_26_H_26_O_11_, exhibiting an exclusive match with procumbenoside L in our database. The fragmentation of the molecular ion of Cpd.**99** at *m/z* 532.1800, as illustrated in Fig. 1a, produced a predominant product ion at *m/z* 378.7631, resulting from the loss of the dimethoxyphenyl group, and another ion at *m/z* 353.0076 due to the loss of the glucose moiety. Consequently, Cpd.**99** was identified as a lignan, namely procumbenoside L. Similarly, Cpd.**112** (t_R_ = 14.85 min) was identified based on its[M + H]^+^ ion at *m/z* 239.0815 (-0.14 ppm). Its molecular formula was deduced as C_14_H_10_N_2_O_2_, demonstrating an exclusive correspondence to 3-(2'-hydroxyphenyl)-4(3*H*)-quinazolinone in our database. Furthermore, Fig. 1b exhibits the fragmentation pattern of the molecular ion of Cpd.**112** at *m/z* 239.0815, revealing characteristic ions at *m/z* 132.0448 resulting from the loss of the hydroxyphenyl group and the oxygen atom at the carbonyl group of the pyrimidine ring. Successive losses of the carbon atom from the benzene ring led to the formation of ions at *m/z* 120.0445 and 108.0437. Therefore, Cpd.**112** was identified as an alkaloid, namely 3-(2'-hydroxyphenyl)-4(3*H*)-quinazolinone.

### Confirmation of constituents using reference standards

**Fig. 2:**
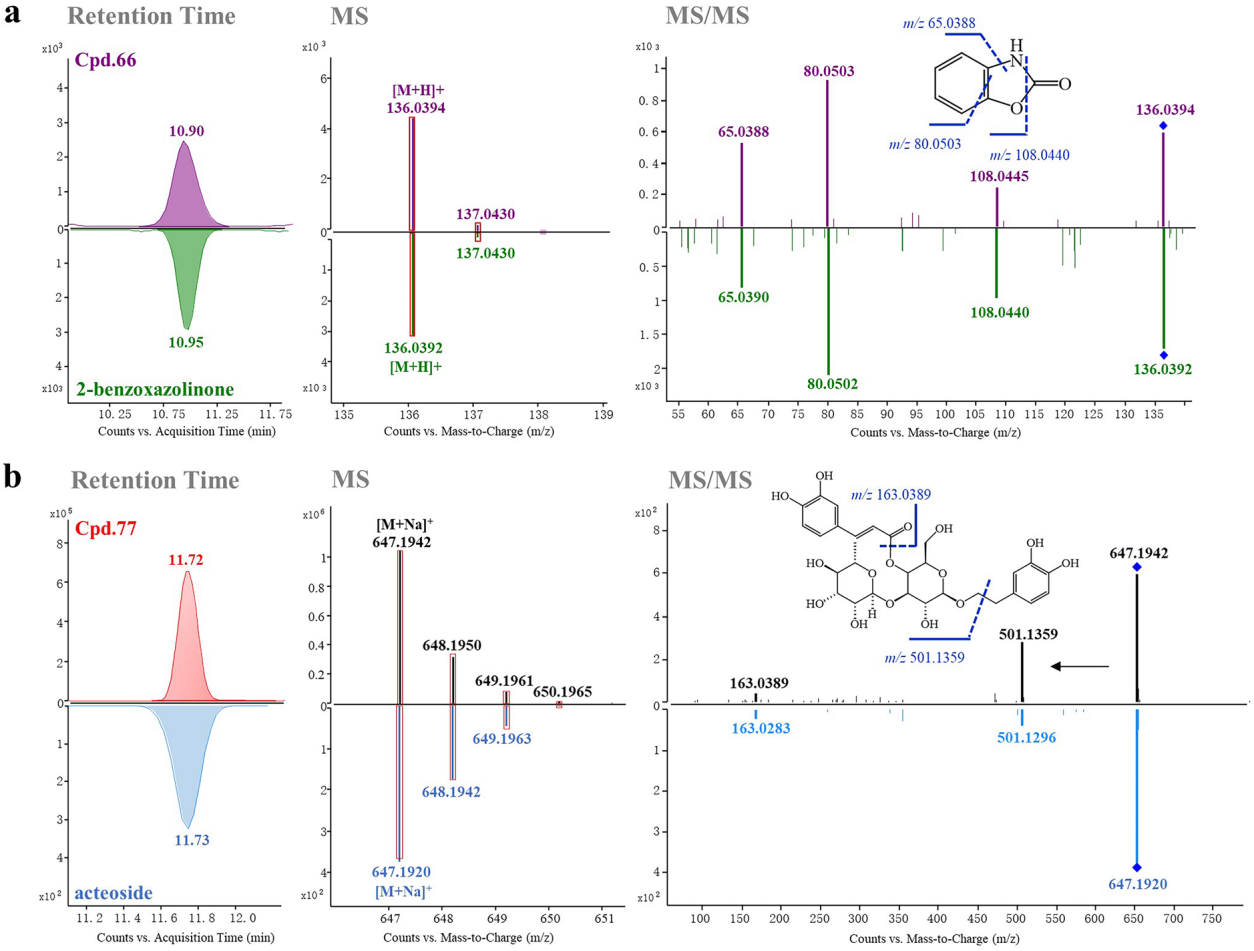
Confirmation of identified constituents by comparing with corresponding reference standards based on retention time, accurate molecular mass, and fragmentation pathway. **a** Cpd.**66** (2-benzoxazolinone) and **b** Cpd.**77** (acteoside)

**Fig. 3:**
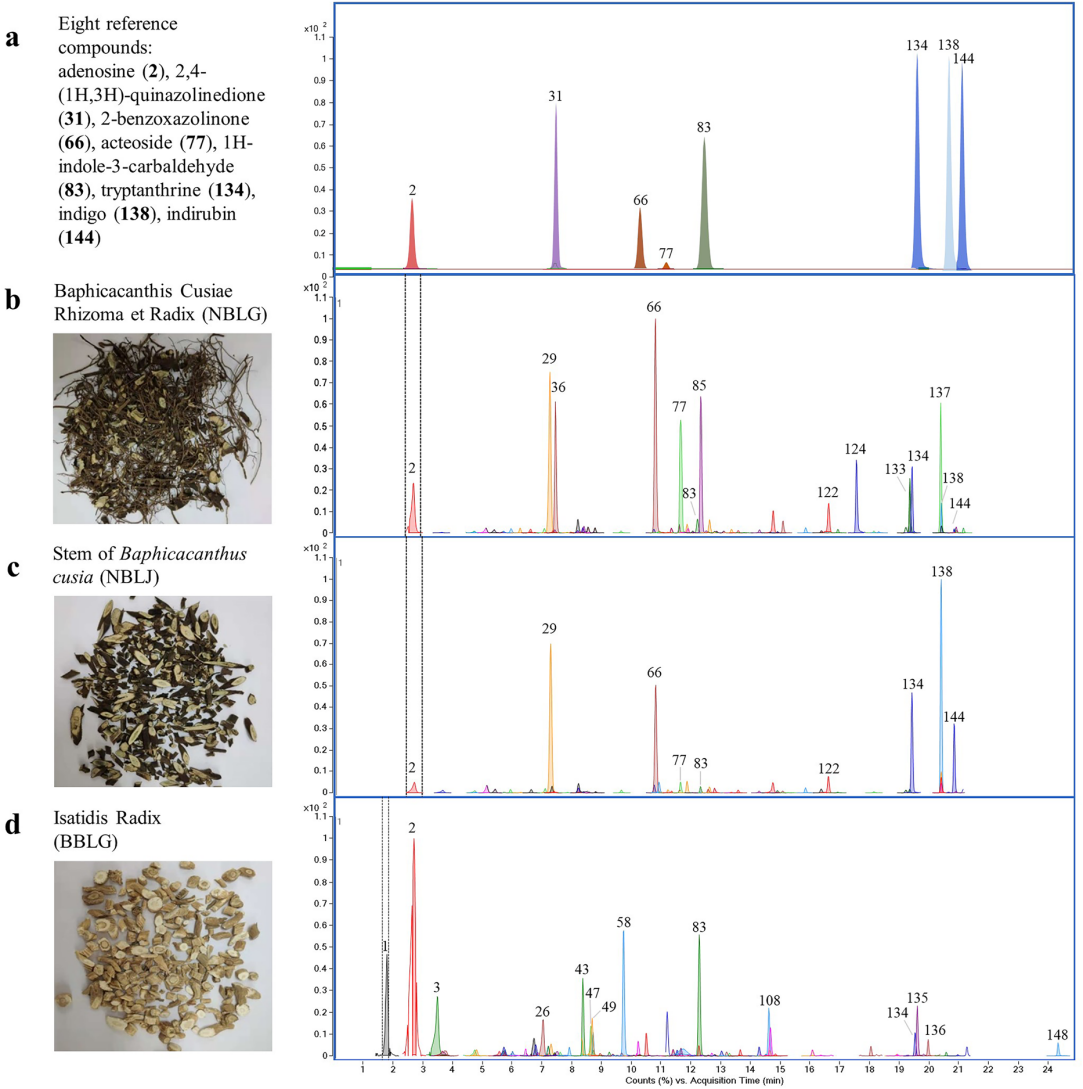
Photographs and extracted compound chromatogram (ECC) of reference standards and samples. **a** Eight reference standards; **b** Baphicacanthis Cusiae Rhizoma et Radix (NBLG); **c** stem of *Baphicacanthus cusia* (NBLJ); and **d** Isatidis Radix (BBLG)

To verify the structures of constituents identified in NBLG, eight reference standards, including adenosine (**2**), 2,4-(1*H*,3*H*)-quinazolinedione (**31**), 2-benzoxazolinone (**66**), acteoside (**77**), 1*H*-indole-3-carbaldehyde (**83**), tryptanthrine (**134**), indigo (**138**), and indirubin (**144**) were further employed for identification (marked with “*” in Additional file 1: Table S1), and their ECCs are presented in Fig. 3a. For instance, as shown in Fig. 2a (upper panel), Cpd.**66** (t_R_ = 10.90 min) was identified with an accurate [M + H]^+^ ion at *m/z* 136.0394 (0.73 ppm), and its molecular formula was determined as C_7_H_5_NO_2_. This formula exclusively matched with 2-benzoxazolinone in our database. The fragmentation of the molecular ion of Cpd.**66** at *m/z* 136.0394 yielded predominant product ions at *m/z* 108.0445 and 80.0503, attributed to the successive loss of CO groups, as well as an ion at *m/z* 65.0388 resulting from the subsequent loss of an NH group. These findings were further validated through a comparison with the retention time, high resolution MS and MS/MS spectrum of reference standards (lower panel of Fig. 2a). Consequently, Cpd.**66** exhibited identical retention time, accurate molecular mass, and fragmentation pathway as 2-benzoxazolinone, confirming its identification as 2-benzoxazolinone.

Likewise, the identification of Cpd.**77** is demonstrated in Fig. 2b (upper panel). Cpd.**77** (t_R_ = 11.72 min) was identified based on an accurate [M + Na]^+^ ion at *m/z* 647.1942 (− 0.62 ppm), and its molecular formula was deduced as C_29_H_36_O_15_. This formula corresponded to acteoside or isoacteoside in our database. Additionally, the fragmentation of the molecular ion of Cpd.**77** at *m/z* 647.1942 yielded predominant product ions at *m/z* 501.1359, attributed to the loss of the hydroxytyrosol group, and *m/z* 163.0389, generated from the caffeic acid moiety. These results were further confirmed through a comparison with the reference standard (lower panel of Fig, 2b). Consequently, Cpd.**77** displayed identical retention time, accurate molecular mass, and fragmentation pathway as acteoside, confirming its identification as acteoside.

### Chemical constituents identified in NBLG

The chemical components identified in NBLG encompass six classes, namely alkaloids, phenylpropanoids, lignans, sesquiterpene lactones, flavonoids, and other compounds. Alkaloids exhibit the highest diversity among the identified components, with a total of 50 alkaloids identified, including 19 indole alkaloids, 13 quinazoline alkaloids, two pyridine alkaloids, one oxazole alkaloid, and other alkaloids. The five most abundant alkaloids found in NBLG were 2-*O*-*β*-D-glucopyranosyl-(2*H*)-1,4-benzoxazin-3(4*H*)-one (**29**), 2-*O*-*β*-D-glucopyranosyl-4-hydroxy-(2*H*)-1,4-benzoxazin-3(4*H*)-one (**36**), 2-benzoxazolinone (**66**), 1,2,3,4-tetrahydro-2-oxo-4-quinolinecarboxamide (**122**), and baphicacanthin A (**124**). Additionally, ten phenylpropanoids, including acteoside (**77**) and its isomer (**85**), were identified in NBLG. Furthermore, NBLG contains four lignans, including 6'-*O*-(1-hydroxy-4-oxocyclohexylacety) acteoside (**133**), two sesquiterpene lactones, one flavonoid, and six other types of compounds. This comprehensive chemical profile of NBLG represents the most extensive analysis to date. Nevertheless, further efforts necessitate additional reference compounds for rigorous characterization.

### Discrimination between NBLG and NBLJ

Due to the scarcity of NBLG resources, NBLJ has been used illegally in the market as a substitute for NBLG. A comprehensive comparison of the chemical components of NBLG and NBLJ can provide pivotal evidence for the reasonable usage of NBLJ. The chemical components in eleven batches of NBLJ, originating from the same whole plant as NBLG, were profiled using the same approach.

A total of 64 compounds were identified in NBLJ, including 55 compounds that were also present in NBLG, while nine compounds (Cpd. **17**, **68**, **98**, **100**, **115**, **117**, **139**, **140**, **142**) were unique in NBLJ. The ECC of the QC sample of NBLJ is shown in Fig. 3c, and the MS and MS/MS data of the identified components are summarized in Additional file 1: Table S1.

**Fig. 4:**
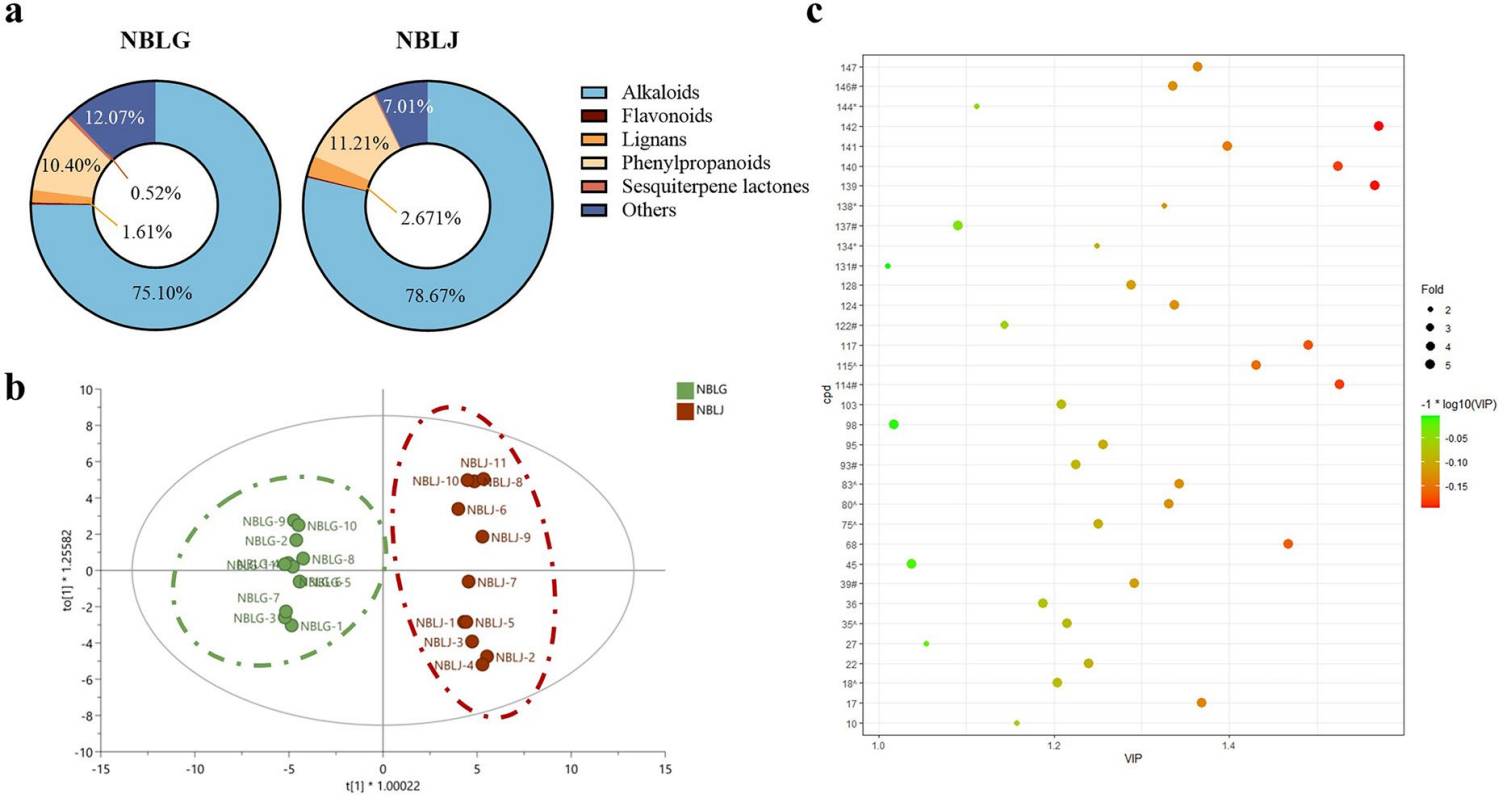
Discrimination between NBLG and NBLJ based on the chemical profiling of eleven batches of *Baphicacanthus cusia* (Nees) Bremek. **a** Comparison of relative percentages of different compound classes within NBLG and NBLJ; **b** OPLS-DA score plot showing the discrimination between NBLG and NBLJ (R^2^X = 0.427, R^2^Y = 0.973, and the Q^2^ = 0.930); **c** the 34 chemical markers (VIP > 1) able to differentiate NBLG from NBLJ

To visualize the overall distribution of chemical constituents in NBLG and NBLJ, the relative abundances of six types of constituents are summarized in Fig. 4a. It can be observed that alkaloids were the most abundant class in both samples, followed by other compounds and phenylpropanoids. Although the chemical components of NBLG and NBLJ are largely similar, some notable differences exist. To identify the chemical markers responsible for distinguishing NBLG from NBLJ, an OPLS-DA analysis was performed. As shown in Fig. 4b, NBLG and NBLJ can be separated into two distinct groups, with an R^2^X value of 0.427, an R^2^Y value of 0.973, and a Q^2^ value of 0.930. A VIP analysis was conducted to identify the components that can differentiate between NBLG and NBLJ, resulting in a total of 34 compounds with VIP > 1 being assigned as markers for the differentiation of NBLG and NBLJ, as demonstrated in Fig. 4c. Notably, all eight of the most significantly different compounds were alkaloids.

In summary, NBLG and NBLJ exhibit high similarity in composition but with significant differences in content. Due to the scarcity of NBLG, NGLJ is sometimes used as a substitute in the market. Therefore, a comprehensive comparison of the chemical constituents of NBLG and NBLJ can provide essential evidence for the appropriate utilization of NBLJ.

### Discrimination between NBLG and BBLG

Chemical components in BBLG were profiled using the same strategy as that used for NBLG. Specifically, the ECC of the QC sample of BBLG is depicted in Fig. 3d. The analysis revealed the identification of 84 compounds in BBLG, with 54 compounds being newly identified in BBLG (as denoted by a “^^^” in Additional file 1: Table S1). Among these identified compounds, only 17 (Cpd. **2**, **7**, **15**, **18**, **21**, **29**, **32**, **35**, **37**, **72**, **75**, **80**, **83**, **102**, **112**, **134**, **144**) were found to be common with NBLG.

**Fig. 5:**
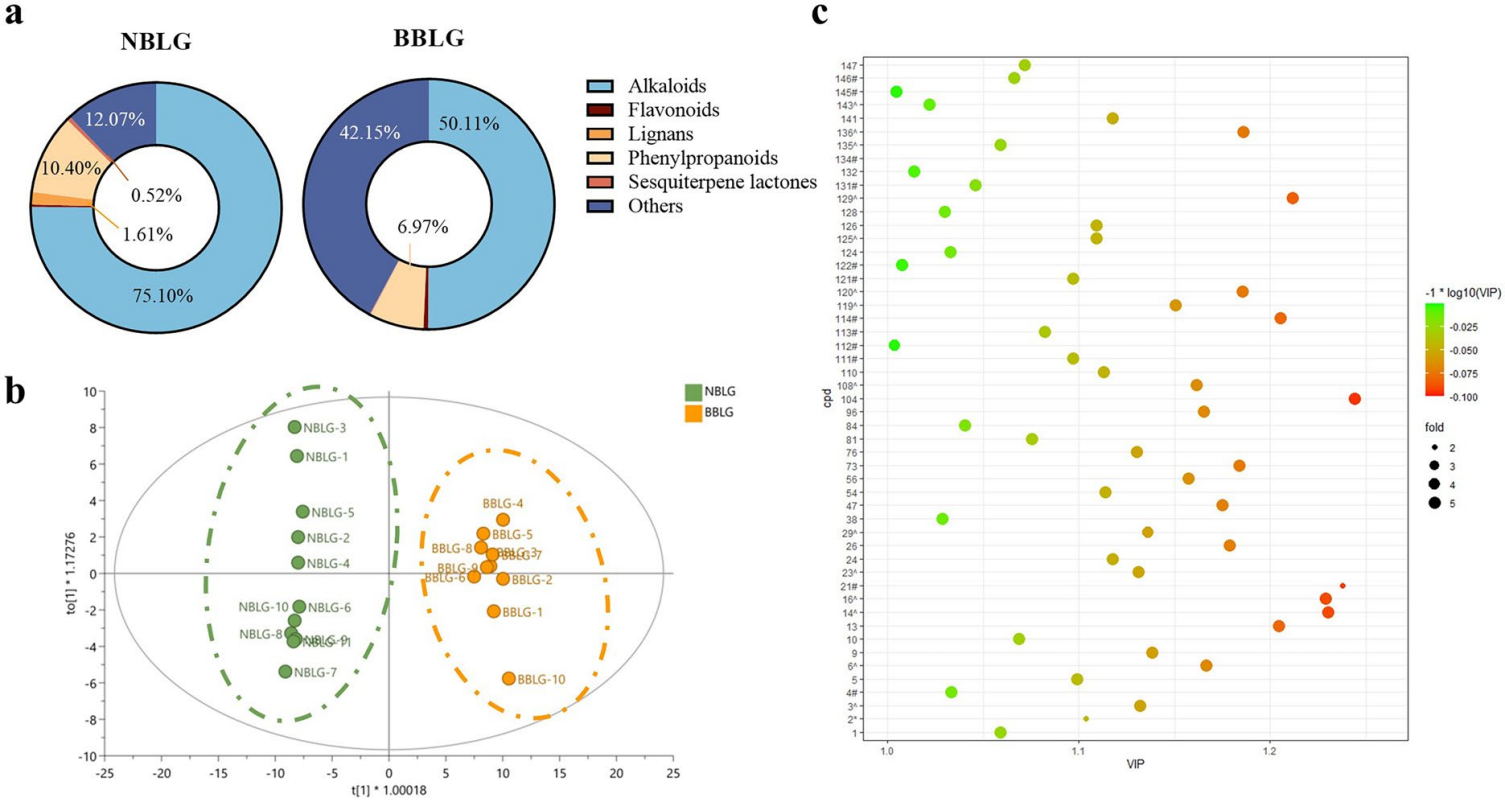
Discrimination between NBLG and BBLG based on the chemical profiling of eleven batches of NBLG and ten batches of BBLG. **a** Comparison of relative percentages of different compound classes within NBLG and BBLG; **b** the OPLS-DA score plot illustrating discrimination between NBLG and BBLG (R^2^X = 0.643, R^2^Y = 0.983, and the Q^2^ = 0.964); **c** the 51 chemical markers (VIP > 1) able to differentiate NBLG from BBLG

Furthermore, the chart presented in Fig. 5a demonstrates a significant difference in the relative percentage of compound classes between NBLG and BBLG. Notably, the percentage of alkaloids in BBLG was found to be significantly lower than that in NBLG. Moreover, the OPLS-DA score plot (Fig. 5b) displayed a clear separation between NBLG and BBLG, with corresponding R^2^X, R^2^Y, and Q^2^ values of 0.643, 0.983, and 0.964, respectively. Based on these findings, a total of 51 compounds with VIP > 1 were identified as potential chemical markers for differentiating NBLG and BBLG (as shown in Fig. 5c).

Collectively, our results demonstrate significant differences between the chemical profiles of NBLG and BBLG, providing chemical evidence for the independent recognition of NBLG and BBLG in the CHP. Importantly, the marked divergence between NBLG and BBLG suggests that BBLG cannot be used as a substitute for NBLG in clinical or manufacturing applications.

### Method validation for HPLC fingerprint analysis

Seven peaks, which collectively accounted for over 90% of the total chromatographic peak area, were selected as characteristic peaks for method validation in the HPLC fingerprint analysis. Among them, peak 4 was identified as the reference peak due to its appropriate retention time and high abundance. To assess the precision, reproducibility, and stability of the HPLC fingerprint method, the RSDs of the relative retention times (RRTs) of the seven characteristic peaks were determined. The RSD values of all the RRTs were found to be below 0.8%, indicating that the established HPLC fingerprint method was repeatable and reliable.

### HPLC fingerprint analysis of NBLG

**Fig. 6:**
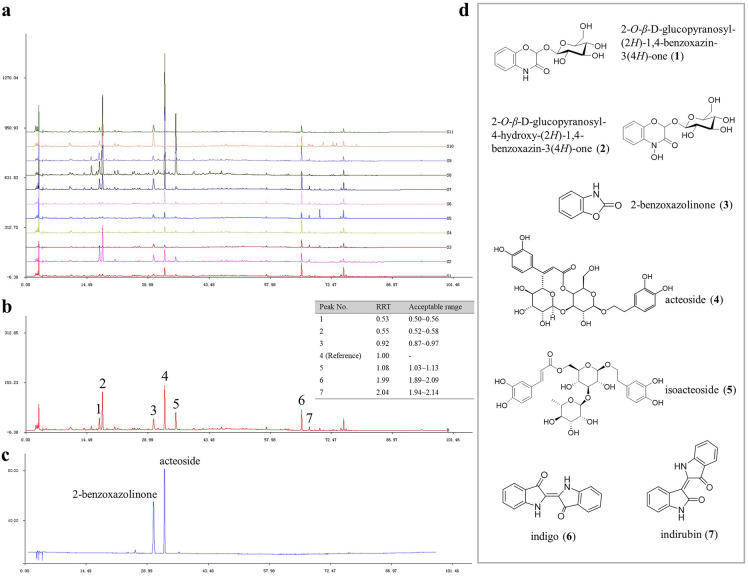
HPLC fingerprint of NBLG. **a** HPLC fingerprint of eleven batches of NBLG; **b** reference chromatogram of eleven batches of NBLG; **c** HPLC chromatogram of two reference standards;** d** chemical structures of the seven characteristic compounds identified in NBLG

The HPLC fingerprints of eleven batches of NBLG were established by selecting and normalizing the retention times of common peaks in the chromatograms, as shown in Fig. 6a. Common peaks with good stability and resolution in all batches were identified, leading to the recognition of seven characteristic peaks in the HPLC fingerprint of NBLG, as depicted in Fig. 6b. The identity of peaks 1 to 7 was confirmed through the employment of commercial standards (Fig. 6c) and UHPLC-Q-TOF–MS analysis as discussed above. The peaks were successively identified as 2-*O*-*β*-D-glucopyranosyl-(2*H*)-1,4-benzoxazin-3(4*H*)-one, 2-*O*-*β*-D-glucopyranosyl-4-hydroxy-(2*H*)-1,4-benzoxazin-3(4*H*)-one, 2-benzoxazolinone, acteoside, isoacteoside, indigo, and indirubin. The RRTs and acceptable ranges (± 5%) of the seven characteristic common peaks of NBLG were provided. The chemical structures of these peaks are demonstrated in Fig. 6d.

HPLC fingerprinting analysis is a widely used and valuable technique for characterizing Traditional Chinese Medicine (TCM) [18]. However, informative HPLC fingerprints for NBLG are limited, with only a few peaks observed in the chromatogram or the identification of limited components [19]. For example, the Hong Kong Chinese Materia Medica Standards (HKCMMS) displayed an NBLG HPLC fingerprint with only three characteristic peaks, including indigo, indirubin, and an unidentified component. Previous literature has reported that the bioactivities of NBLG are derived from various components. Therefore, in this study, we established an HPLC fingerprint of NBLG that included seven components from two distinct classes of components (alkaloids and phenylpropanoids). This comprehensive fingerprint can be more reasonably applied in the quality assessment of NBLG.

### Selection of chemical markers for the quality control of NBLG

The current edition of the CHP does not provide a specification for the content of marker compounds in NBLG, which is crucial for ensuring the quality control of Chinese medicines. The CHP guidelines suggest that ideal marker compounds should be abundant, easily obtainable, exhibit bioactivity, and have a specific presence. However, for NBLG, previously proposed marker compounds such as indigo, indirubin, tryptanthrin, adenosine, and 4(3*H*)-quinazolione have relatively low content in NBLG (less than 1/10000) [20]. Moreover, indigo, one of the marker compounds, is quite unstable and needs to be detected within 8 h. As shown in our previous chemical study [21] and the profiling conducted in the current study, the chemical components of NBLG are complex and diverse, including alkaloids, flavonoids, organic acids, glycosides, pentacyclic triterpenes, amino acids, anthraquinones, carbohydrate, and sterols. Therefore, selecting a suitable marker compound that consistently exists in NBLG with relatively high abundance is essential for the establishment of an assay method.

**Fig. 7:**
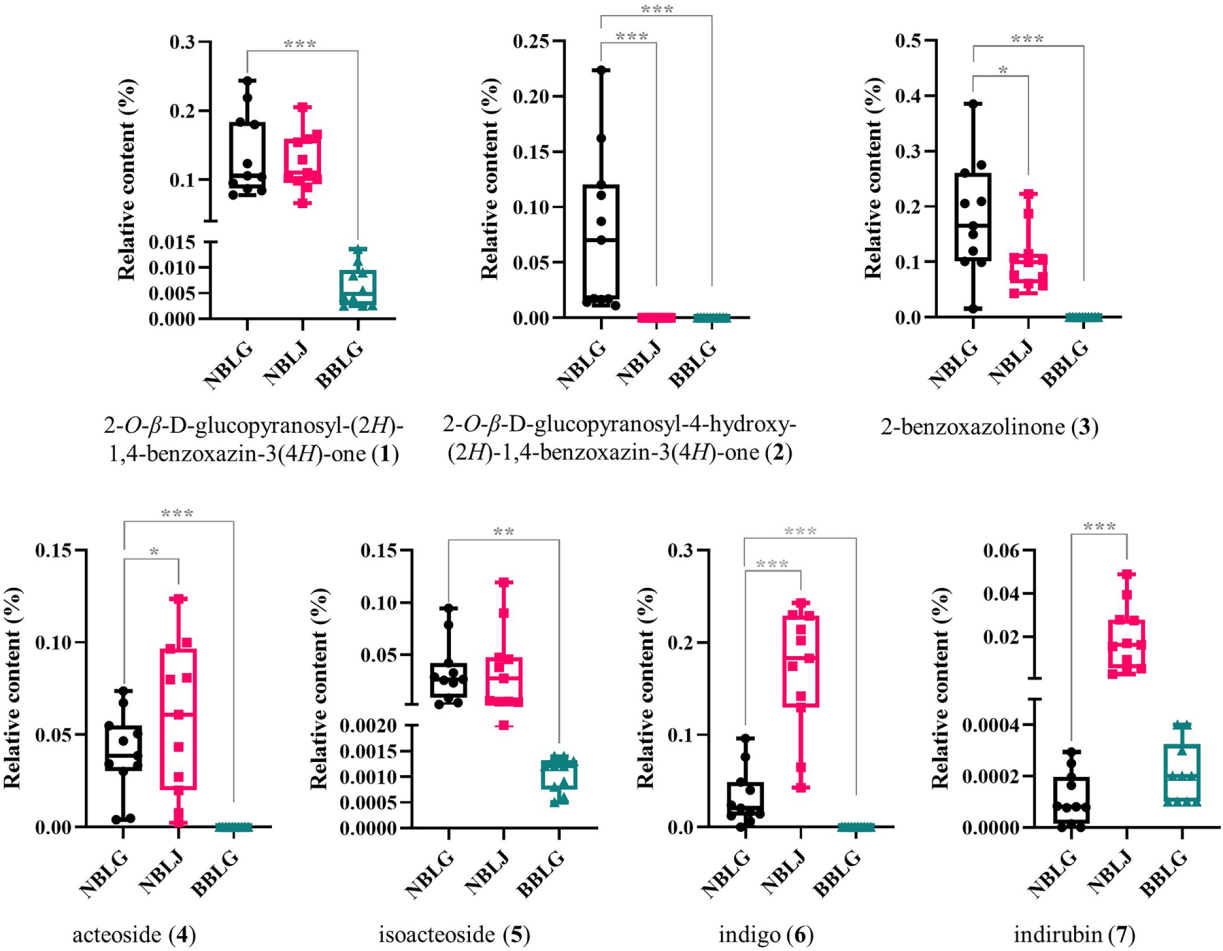
Relative content (%) of the seven characteristic compounds within NBLG, NBLJ, and BBLG. Statistical significance indicated by *: *P*-value < 0.05, **: *P*-value < 0.01, and ***: *P*-value < 0.001

In this study, an HPLC fingerprint of NBLG was established, revealing seven common peaks that were identified as characteristic components of NBLG and proposed as ideal chemical markers. Four of these components, namely 2-*O*-*β*-D-glucopyranosyl-4-hydroxy-(2*H*)-1,4-benzoxazin-3(4*H*)-one, 2-benzoxazolinone, acteoside, and indigo, were not detected in BBLG. Furthermore, significant differences were observed in their content between NBLG and NBLJ (Fig. 7), suggesting their potential as effective markers for distinguishing NBLG from NBLJ and BBLG. Nevertheless, the instability of indigo and the commercial unavailability of 2-*O*-*β*-D-glucopyranosyl-4-hydroxy-(2*H*)-1,4-benzoxazin-3(4*H*)-one render them less ideal markers. In contrast, 2-benzoxazolinone and acteoside are considered preferable due to their commercial availability and relative abundance. Additionally, 2-benzoxazolinone has been found to exhibit anti-influenza activity [22], while acteoside has displayed antioxidant and anti-inflammatory activity [23]. These observations suggest that these two compounds could potentially serve as active constituents of NBLG, making them suitable candidates as ideal chemical markers for NBLG.

### Method validation for HPLC quantitative analysis

Validation was performed for the simultaneous determination of 2-benzoxazolinone and acteoside in NBLG. The calibration curves for both compounds exhibited excellent linearity across the test range, with R^2^ values exceeding 0.9999, as shown in Table 2. The LOD and LOQ for acteoside were determined to be 0.42 µg/mL and 0.85 µg/mL, respectively, while those for 2-benzoxazolinone were 0.43 µg/mL and 0.88 µg/mL, respectively. The precision, repeatability, and stability of both compounds exhibited RSD values below 2%. To assess the recovery, the found amounts of 2-benzoxazolinone and acteoside were in the range of 447.18 to 458.05 µg and 754.48 to 770.86 µg, respectively, resulting in mean recoveries of 102.7% and 97.2%, respectively, with both RSD values below 1%. All parameters met the acceptance criteria, indicating the reliability and accuracy of the method for determining 2-benzoxazolinone and acteoside in NBLG.

**Table 2:** Regression equation, linear range, limit of detection (LOD), limit of quantification (LOQ), precision, repeatability, stability, and recovery of 2-benzoxazolinone and acteoside in NBLG

	Compound	
	2-benzoxazolinone	acteoside
Regression equation	y = 17.67x + 15.04	y = 15.73x-19.73
Linear range (µg/mL)	1.59 − 1016.00	1.73 − 1110.00
Correlation coefficient, *R*^2^	1.0000	0.9999
LOD (µg/mL)	0.43	0.42
LOQ (µg/mL)	0.88	0.85
Precision (RSD%, *n* = 6)	0.60	0.80
Repeatability (RSD%, *n* = 6)	0.90	1.20
Stability (RSD%, *n* = 9)	1.60	1.00
Recovery (mean; RSD%, *n* = 6)	102.70; 0.60	97.20; 0.90

### Quantitative determination of the two chemical markers

The developed HPLC method was employed to quantify the content of 2-benzoxazolinone and acteoside in eleven batches of NBLG. As shown in Table 3, the content of 2-benzoxazolinone in NBLG ranged from 61.90 to 548.70 μg/g, with a corresponding content range in dry weight of 0.007–0.062%, and a mean value of 0.023%. For acteoside, its content in NBLG varied from 47.20 to 2219.30 μg/g, with a corresponding content range in dry weight of 0.005–0.546% and a mean value of 0.102%. It is important to note that our findings revealed significant fluctuations in the content of 2-benzoxazolinone and acteoside among different batches of NBLG. Despite standardized processing and storage conditions for all samples, the wide variations among batches are likely attributed to the diverse growth environments and harvesting seasons of the NBLG samples [24]. These influential factors necessitate further investigation in future studies.

**Table 3:** Contents of 2-benzoxazolinone and acteoside in eleven batches of NBLG (*n* = 2)

Batch no	Content (µg/g)		Water content (%)	Content in dry weight (%)	
	2-benzoxazolinone	acteoside		2-benzoxazolinone	acteoside
NBLG-1	61.90	341.00	11.90	0.007	0.039
NBLG-2	107.60	100.60	10.80	0.012	0.011
NBLG-3	70.90	247.50	11.70	0.008	0.028
NBLG-4	129.50	261.90	10.70	0.014	0.029
NBLG-5	62.90	537.60	10.30	0.007	0.060
NBLG-6	425.10	776.20	9.20	0.047	0.085
NBLG-7	172.80	4805.90	11.90	0.020	0.546
NBLG-8	187.00	2219.30	12.10	0.021	0.252
NBLG-9	548.70	69.20	12.20	0.062	0.008
NBLG-10	246.50	47.20	14.00	0.029	0.005
NBLG-11	234.30	499.50	12.50	0.027	0.057
Mean				0.023	0.102

Based on these results, we propose a new quality control standard for NBLG, which specifies that the limit of content in dry weight for both 2-benzoxazolinone and acteoside should not be less than 0.010%. Currently, there are no explicit regulation regarding the component content of NBLG, and the content of 50% ethanol extract remains the primary indicator of NBLG’s quality [16]. Therefore, our proposed standard would provide a crucial benchmark for assessing the quality of NBLG.

## Conclusion

In the present study, we utilized UHPLC Q-TOF–MS to comprehensively profile the chemical constituents of NBLG. Using a personal database, we identified 73 compounds belonging to six distinct classes, with alkaloids being the most diverse and abundant. This study provides the most comprehensive chemical information to date on NBLG, with 36 compounds identified for the first time. Additionally, we compared the chemical components of NBLG to its two adulterant species, NBLJ and BBLG. Although NBLG and NBLJ exhibited high compositional similarities, there were significant differences in their content. In contrast, BBLG had a markedly different chemical profile from NBLG. These findings provide crucial chemical evidence for the independent identification and appropriate utilization of NBLG, NBLJ, and BBLG in clinical or manufacturing applications. Moreover, we established an informative HPLC fingerprint of NBLG comprising seven characteristic peaks that can be used for quality assessment. Notably, 2-benzoxazolinone and acteoside were identified as potential active constituents of NBLG and proposed as ideal chemical markers due to their commercial availability and relative abundance. Finally, we propose a new quality control standard for NBLG, which stipulates that the limit of content in dry weight for both 2-benzoxazolinone and acteoside should not be less than 0.010%. These findings provide valuable information for the authentication and quality control of NBLG and its related products and lay a foundation for further research on the pharmacological activities and mechanisms of NBLG. The study also highlights the importance of comprehensive chemical profiling to identify potential substitutions and adulterations, particularly in cases where the original source is scarce or unavailable.

## Supplementary Information

**Additional file 1: Table S1.** Characterization of chemical components in root and rhizome (NBLG), as well as stem (NBLJ) of *Baphicacanthus cusia* (Nees) Bremek, and root of* Isatis indigotica* Fort. (BBLG)

## Data Availability

The data in this study are available from the corresponding author upon reasonable request.

## Abbreviations

CHP: Chinese Pharmacopoeia
HPLC: High-performance liquid chromatography
UHPLC-Q-TOF-MS: Ultra-high performance liquid chromatography coupled with quadrupole-time-of-flight mass spectrometry
MS: Mass spectrometry
LOD: Limit of detection
LOQ: Limit of quantitation
OPLS-DA: Orthogonal partial least squares discriminant analysis
ECC: Extracted compound chromatogram
RSD: Relative standard deviations
