# Supplementary material for: Discrimination of Baphicacanthis Cusiae Rhizoma et radix and its adulterant species and establishment of an assay method for quality control
Source: Chin Med. 2023 Jun 2;18:65. doi: 10.1186/s13020-023-00777-x (PMC10234792; doi:10.1186/s13020-023-00777-x)
Supplement: Supplementary file 1 — Additional file 1: Table S1. Characterization of chemical components in root and rhizome (NBLG), as well as stem (NBLJ) of Baphicacanthus cusia (Nees) Bremek, and root of Isatis indigotica Fort. (BBLG) [file 13020_2023_777_MOESM1_ESM.docx]

**Discrimination of Baphicacanthis Cusiae Rhizoma et Radix and its adulterant species and establishment of an assay method for quality control**

Yue Li^1^, Qiong-Xi Yu^1^, Lee-Fong Yau^1^, Guo-Kai Huang^4^, Jing-Guang Lu^1^, Xiao-Xiao Liu^4^，Zhi-Hong Jiang^1,*^, Jing-Rong Wang^2,3,1,*^

^1^ *State Key Laboratory of Quality Research in Chinese Medicines, Macau Institute for Applied Research in Medicine and Health, Macau University of Science and Technology, Taipa, 999078, Macao, China*

^2^ *State Key Laboratory of Dampness Syndrome of Chinese Medicine, The Second Affiliated Hospital of Guangzhou University of Chinese Medicine, Guangzhou, 510000, China*

^3^ *Guangdong-Hong Kong-Macau Joint Lab on Chinese Medicine and Immune Disease Research, Guangzhou, 510000, China*

^4^ *Guangdong Institute for Drug Control, Guangzhou,* *510663, China*

**Corresponding author: Jing-Rong Wang Ph.D**

Professor

The Second Affiliated Hospital of Guangzhou University of Chinese Medicine

Guangzhou, China.

Tel: +86-20-39318473

E-mail: jrwang@gzucm.edu.cn

**Table S1** Characterization of chemical components in root and rhizome (NBLG), as well as stem (NBLJ) of *Baphicacanthus cusia* (Nees) Bremek, and root of *Isatis indigotica* Fort. (BBLG)

| No. | t_R_ (min) | Molecular formula | Molecular ion | Theoretical mass (*m/z*) | Diff (ppm) | Measured Mass (m/z) | Fragmentations ions (*m/z*) | Name | Chemical Type | Presence in | | |
| --- | --- | --- | --- | --- | --- | --- | --- | --- | --- | --- | --- | --- |
|  |  |  |  |  |  |  |  |  |  | NBLG | NBLJ | BBLG |
| 1 | 1.80 | C_5_H_7_NOS | [M+H]^+^ | 130.0321 | 3.03 | 130.0325 |  | epigoitrin | A |  |  | 🗸 |
| 2* | 2.71 | C_10_H_13_N_5_O_4_ | [M+H]^+^ | 268.1040 | -1.70 | 268.1035 | 250.9084, 210.9074, 136.0626 | adenosine | O | 🗸 | 🗸 | 🗸 |
| 3^^^ | 3.51 | C_9_H_7_NO | [M+H]^+^ | 146.0600 | -0.19 | 146.0600 |  | isomer of 83 | A |  |  | 🗸 |
| 4^#^ | 3.65 | C_12_H_12_N_2_O_3_ | [M+H]^+^ | 233.0921 | 0.81 | 233.0923 | 171.0922, 84.9067 | adhavasinone | A | 🗸 | 🗸 |  |
| 5 | 3.66 | C_10_H_10_N_2_O_3_ | [M+H]^+^ | 207.0764 | 2.54 | 207.0769 |  | 2,3-dihydro-3-hydroxy-2-oxo-1*H*-indole-3-acetamide | O |  |  | 🗸 |
| 6^^^ | 3.81 | C_9_H_7_NO_2_ | [M+H]^+^ | 162.0550 | -2.17 | 162.0546 |  | 3-carboxyindole | O |  |  | 🗸 |
| 7^#^ | 4.75 | C_12_H_12_N_2_O_2_ | [M+H]^+^ | 217.0972 | -0.03 | 217.0972 | 199.0159, 145.0776, 114.9838, 84.9607 | cycloanthranilylproline | A | 🗸 | 🗸 | 🗸 |
| 8^#^ | 4.78 | C_11_H_12_N_2_O_2_ | [M+H]^+^ | 205.0972 | 4.44 | 205.0981 | 190.0729, 162.0926, 132.8858 | vasicinol | A | 🗸 | 🗸 |  |
| 9 | 4.84 | C_8_H_7_NO_2_ | [M+H]^+^ | 150.0550 | 3.36 | 150.0555 |  | 5-hydroxy-2-indolinone | A |  |  | 🗸 |
| 10 | 5.16 | C_9_H_7_NO | [M+H]^+^ | 146.0600 | 5.19 | 146.0608 | 128.0485, 118.0634, 101.0386, 91.0539, 77.0379, 65.0377, 55.9343 | isomer of 83 | A | 🗸 | 🗸 |  |
| 11 | 5.43 | C_9_H_7_NO | [M+H]^+^ | 146.0600 | -0.49 | 146.0599 | 128.0485, 118.0634, 101.0386, 91.0539, 77.0379, 65.0377, 55.9343 | isomer of 83 | A | 🗸 | 🗸 |  |
| 12 | 5.60 | C_14_H_15_NO_6_ | [M+NH_4_]^+^ | 311.1238 | -0.21 | 311.1237 |  | isatan B | A |  |  | 🗸 |
| 13 | 5.75 | C_10_H_10_N_2_O_3_ | [M+H]^+^ | 207.0764 | -0.78 | 207.0762 |  | isomer of 5 | O |  |  | 🗸 |
| 14^^^ | 5.75 | C_9_H_7_NO_2_ | [M+H]^+^ | 162.0550 | 0.30 | 162.0550 |  | isomer of 6 | O |  |  | 🗸 |
| 15^#^^ | 5.76 | C_11_H_12_N_2_O_2_ | [M+H]^+^ | 205.0972 | -1.48 | 205.0969 | 179.0993, 130.8888, 102.9971 | isomer of 8 | A | 🗸 | 🗸 | 🗸 |
| 16^^^ | 5.80 | C_9_H_7_NO | [M+H]^+^ | 146.0600 | -1.36 | 146.0598 |  | isomer of 83 | A |  |  | 🗸 |
| 17 | 5.94 | C_9_H_8_N_2_O | [M+H]^+^ | 161.0709 | -3.51 | 161.0703 |  | 2-methylquinazolin-4-ol | A |  | 🗸 |  |
| 18^^^ | 6.01 | C_9_H_7_NO | [M+H]^+^ | 146.0600 | 1.46 | 146.0602 |  | isomer of 83 | A | 🗸 |  | 🗸 |
| 19 | 6.30 | C_14_H_17_NO_9_ | [M+Na]^+^ | 366.0796 | -1.02 | 366.0792 | 204.0267, 185.0427, 136.9284 | isomer of 36 | A | 🗸 | 🗸 |  |
| 20 | 6.31 | C_10_H_20_O_6_ | [M+NH_4_]^+^ | 254.1598 | 2.34 | 254.1604 |  | butyl glycoside *β*-D-tagatopyranoside | O |  |  | 🗸 |
| 21^#^ | 6.65 | C_10_H_8_N_2_O_2_ | [M+H]^+^ | 189.0659 | -3.17 | 189.0653 | 128.9441, 105.0742, 98.9796 | 2,3-dihydro-4-hydroxy-2-oxo-1*H*-indole-3-acetonitrile | O | 🗸 | 🗸 | 🗸 |
| 22 | 6.65 | C_15_H_10_O_3_ | [M+Na]^+^ | 261.0522 | 4.52 | 261.0534 | 224.8953, 204.8943, 160.9186 | 1-hydroxy-3-methyl-9,10-anthraquinone | O | 🗸 |  |  |
| 23^^^ | 6.81 | C_9_H_7_NO | [M+H]^+^ | 146.0600 | 1.87 | 146.0603 |  | isomer of 83 | A |  |  | 🗸 |
| 24 | 6.82 | C_10_H_10_N_2_O_2_ | [M+Na]^+^ | 213.0634 | 1.79 | 213.0638 |  | isomer of 122 | A |  |  | 🗸 |
| 25^^^ | 6.87 | C_14_H_17_NO_8_ | [M+Na]^+^ | 350.0846 | 1.15 | 350.0850 |  | isomer of 29 | A |  |  | 🗸 |
| 26 | 7.06 | C_16_H_18_N_2_O_6_ | [M+Na]^+^ | 357.1057 | -9.16 | 357.1024 |  | cappariloside | A |  |  | 🗸 |
| 27 | 7.15 | C_8_H_6_N_2_O | [M+H]^+^ | 147.0553 | 1.78 | 147.0556 | 120.0450, 104.0511, 92.0501 | 4(3*H*)-quinazolinone | A | 🗸 | 🗸 |  |
| 28^^^ | 7.22 | C_14_H_11_NO_2_ | [M+NH_4_]^+^ | 243.1128 | 4.12 | 243.1138 |  | 2-benzyloxybenzoxazole | A |  |  | 🗸 |
| 29^^^ | 7.31 | C_14_H_17_NO_8_ | [M+Na]^+^ | 350.0846 | 4.11 | 350.0860 | 304.0411, 250.0287, 188.0367 | 2-*O*-*β*-D-glucopyranosyl-(2*H*)-1,4-benzoxazin-3(4*H*)-one | A | 🗸 | 🗸 | 🗸 |
| 30 | 7.32 | C_14_H_17_NO_6_ | [M+Na]^+^ | 318.0948 | 7.67 | 318.0972 |  | indican | A |  |  | 🗸 |
| 31* | 7.38 | C_8_H_6_N_2_O_2_ | [M+H]^+^ | 163.0502 | 3.93 | 163.0508 | 136.0437, 119.0410, 108.0487, 80.0525, 65.0408, 53.0404 | 2,4-(1*H*,3*H*)-quinazolinedione | A | 🗸 | 🗸 |  |
| 32^#^^ | 7.41 | C_7_H_11_NO | [M+H]^+^ | 126.0913 | 1.16 | 126.0914 | 109.0697, 81.0729, 58.0677, 55.0567 | 6-acetyl-1,2,3,4-tetrahydropyridine | A | 🗸 | 🗸 | 🗸 |
| 33^^^ | 7.44 | C_14_H_11_NO_2_ | [M+NH_4_]^+^ | 243.1128 | 2.77 | 243.1135 |  | isomer of 28 | A |  |  | 🗸 |
| 34 | 7.47 | C_12_H_12_N_2_O_2_ | [M+H]^+^ | 217.0972 | 2.48 | 217.0977 |  | isomer of 7 | A |  |  | 🗸 |
| 35^^^ | 7.48 | C_21_H_26_O_10_ | [M+H]^+^ | 439.1599 | -2.61 | 439.1588 | 398.0164, 262.0039, 203.9968, 170.0230 | 13-hydroxy indigotide A | O | 🗸 |  | 🗸 |
| 36 | 7.50 | C_14_H_17_NO_9_ | [M+Na]^+^ | 366.0796 | -0.10 | 366.0796 | 231.0412, 185.0361 | 2-*O*-*β*-D-glucopyranosyl-4-hydroxy-(2*H*)-1,4-benzoxazin-3(4*H*)-one | A | 🗸 |  |  |
| 37^#^^ | 7.89 | C_11_H_10_N_2_O_2_ | [M+H]^+^ | 203.0815 | -3.40 | 203.0808 | 185.0706, 148.0398, 130.0640 | vasicinone | A | 🗸 | 🗸 | 🗸 |
| 38 | 7.92 | C_27_H_30_O_15_ | [M+H]^+^ | 595.1657 | -0.94 | 595.1651 |  | saponarin | F |  |  | 🗸 |
| 39^#^ | 8.01 | C_8_H_9_NO | [M+H]^+^ | 136.0757 | -3.45 | 136.0752 | 121.0872, 98.9759 | 1-(2-pyridinyl)-1-propanone | A | 🗸 |  |  |
| 40^#^ | 8.26 | C_10_H_10_N_2_O_2_ | [M+H]^+^ | 191.0815 | -0.90 | 191.0813 | 147.0548, 132.9738 | isomer of 122 | A | 🗸 | 🗸 |  |
| 41^#^ | 8.28 | C_8_H_5_NO_2_ | [M+H]^+^ | 148.0393 | 0.42 | 148.0394 | 120.0446, 93.0337, 65.0386, 51.0221 | isatin | A | 🗸 | 🗸 |  |
| 42^#^ | 8.34 | C_15_H_17_NO_6_ | [M+NH_4_]^+^ | 325.1394 | -0.18 | 325.1393 | 307.1290, 191.0811, 163.0365, 81.0352 | dihydroascorbigen | A | 🗸 | 🗸 |  |
| 43^^^ | 8.38 | C_11_H_16_O_3_ | [M+Na]^+^ | 219.0992 | -4.82 | 219.0981 |  | loliolide | S |  |  | 🗸 |
| 44^#^ | 8.43 | C_10_H_10_N_2_O_2_ | [M+H]^+^ | 191.0815 | -3.67 | 191.0808 | 147.0548, 132.9738 | isomer of 122 | A | 🗸 | 🗸 |  |
| 45 | 8.48 | C_8_H_5_NO_3_ | [M+H]^+^ | 164.0342 | -3.21 | 164.0337 | 136.0387, 119.0350, 108.0457, 80.0492, 65.0381, 53.0385 | isatoic anhydride | A | 🗸 |  |  |
| 46^#^ | 8.59 | C_12_H_12_N_2_O_2_ | [M+H]^+^ | 217.0972 | -4.28 | 217.0963 | 199.0827, 80.0497 | isomer of 7 | A | 🗸 | 🗸 |  |
| 47 | 8.66 | C_10_H_9_NO_2_ | [M+H]^+^ | 176.0706 | -5.57 | 176.0696 |  | 1-methoxyindole-3-carboxaldehyde | A |  |  | 🗸 |
| 48^^^ | 8.71 | C_33_H_46_O_17_ | [M+Na]^+^ | 737.2627 | -0.02 | 737.2627 |  | isomer of 128 | L |  |  | 🗸 |
| 49^^^ | 8.71 | C_8_H_11_NO | [M+Na]^+^ | 160.0733 | 6.37 | 160.0743 |  | thomandersine | A |  |  | 🗸 |
| 50^#^ | 8.77 | C_15_H_17_NO_6_ | [M+NH_4_]^+^ | 325.1394 | -2.90 | 325.1385 | 307.1295, 191.0813, 163.0388 | isomer of 42 | A | 🗸 | 🗸 |  |
| 51^#^ | 8.84 | C_12_H_12_N_2_O_2_ | [M+H]^+^ | 217.0972 | 0.33 | 217.0973 | 199.0858, 182.0607, 108.0427, 80.0497 | isomer of 7 | A | 🗸 | 🗸 |  |
| 52^^^ | 8.96 | C_31_H_40_O_15_ | [M+Na]^+^ | 675.2259 | -1.27 | 675.2250 |  | isomartynoside | P |  |  | 🗸 |
| 53^^^ | 9.28 | C_15_H_17_NO_6_ | [M+H]^+^ | 308.1129 | 0.46 | 308.1130 |  | isomer of 42 | A |  |  | 🗸 |
| 54 | 9.39 | C_27_H_30_O_15_ | [M+H]^+^ | 595.1657 | -1.87 | 595.1646 |  | isomer of 38 | F |  |  | 🗸 |
| 55^^^ | 9.57 | C_10_H_10_N_2_O | [M+H]^+^ | 175.0866 | -5.44 | 175.0856 |  | *N*-ethyl-quinazolin-4(3*H*)-one | A |  |  | 🗸 |
| 56 | 9.69 | C_21_H_16_N_4_O | [M+H]^+^ | 341.1397 | -2.59 | 341.1388 |  | *α*7-(1*H*-Indol-3-yl)-4-methoxy-1*H*-indole-3,7-diacetonitrile | A |  |  | 🗸 |
| 57^#^ | 9.72 | C_10_H_10_N_2_O_3_ | [M+Na]^+^ | 229.0584 | -2.06 | 229.0579 | 140.9180, 87.0571 | isomer of 5 | O | 🗸 | 🗸 |  |
| 58^^^ | 9.74 | C_11_H_10_N_2_O | [M+H]^+^ | 187.0866 | 4.87 | 187.0875 |  | 2,3-trimethylene-4-quinazolone | A |  |  | 🗸 |
| 59^^^ | 10.10 | C_37_H_46_O_18_ | [M+Na]^+^ | 801.2576 | -1.11 | 801.2567 |  | isomer of 133 | L |  |  | 🗸 |
| 60^^^ | 10.24 | C_11_H_12_N_2_O | [M+H]^+^ | 189.1022 | 9.36 | 189.1040 |  | peganine | A |  |  | 🗸 |
| 61^^^ | 10.27 | C_37_H_46_O_18_ | [M+Na]^+^ | 801.2576 | -1.29 | 801.2566 |  | isomer of 133 | L |  |  | 🗸 |
| 62^^^ | 10.50 | C_11_H_16_O_3_ | [M+Na]^+^ | 219.0992 | 4.41 | 219.1002 |  | isomer of 43 | S |  |  | 🗸 |
| 63^^^ | 10.59 | C_37_H_46_O_18_ | [M+Na]^+^ | 801.2576 | -0.68 | 801.2571 |  | isomer of 133 | L |  |  | 🗸 |
| 64^#^ | 10.83 | C_12_H_12_N_2_O_3_ | [M+H]^+^ | 233.0921 | -2.10 | 233.0916 | 147.0557, 87.0439 | isomer of 4 | A | 🗸 | 🗸 |  |
| 65^^^ | 10.85 | C_37_H_46_O_18_ | [M+Na]^+^ | 801.2576 | -3.46 | 801.2548 |  | isomer of 133 | L |  |  | 🗸 |
| 66* | 10.88 | C_7_H_5_NO_2_ | [M+H]^+^ | 136.0393 | -1.45 | 136.0391 | 108.0444, 93.0329, 80.0492, 65.0386, 53.0382 | 2-benzoxazolinone | A | 🗸 | 🗸 |  |
| 67 | 10.99 | C_11_H_16_O_3_ | [M+H]^+^ | 197.1172 | 0.24 | 197.1172 | 179.1052, 107.0794 | isomer of 43 | S | 🗸 | 🗸 |  |
| 68 | 10.99 | C_8_H_5_NO_2_ | [M+H]^+^ | 148.0393 | -6.54 | 148.0383 |  | isomer of 41 | A |  | 🗸 |  |
| 69^#^ | 11.29 | C_17_H_17_NO_9_ | [M+H]^+^ | 380.0976 | -4.24 | 380.0960 |  | isatan A | A | 🗸 | 🗸 |  |
| 70^^^ | 11.35 | C_28_H_38_O_13_ | [M+NH_4_]^+^ | 600.2651 | -0.86 | 600.2646 |  | alangilignoside C | L |  |  | 🗸 |
| 71^^^ | 11.35 | C_16_H_10_N_2_O_3_ | [M+H]^+^ | 279.0764 | -9.34 | 279.0738 |  | OH-indirubin | A |  |  | 🗸 |
| 72^^^ | 11.41 | C_20_H_22_O_8_ | [M+Na]^+^ | 413.1207 | -3.04 | 413.1194 |  | guaiacylglycerol-*β*-ferulic acid ether | P | 🗸 | 🗸 | 🗸 |
| 73 | 11.51 | C_16_H_18_N_2_O_5_S | [M+Na]^+^ | 373.0829 | -2.10 | 373.0821 |  | indole-3-acetonitrile-2-*S*-*β*-D-glucopyranoside | A |  |  | 🗸 |
| 74^^^ | 11.55 | C_11_H_16_O_3_ | [M+Na]^+^ | 219.0992 | 3.53 | 219.1000 |  | isomer of 43 | S |  |  | 🗸 |
| 75^^^ | 11.67 | C_20_H_22_O_8_ | [M+Na]^+^ | 413.1207 | 0.48 | 413.1209 | 237.1025 | isomer of 72 | P | 🗸 |  | 🗸 |
| 76 | 11.70 | C_22_H_22_O_11_ | [M+Na]^+^ | 485.1054 | -2.32 | 485.1043 |  | isoscoparin | F |  |  | 🗸 |
| 77* | 11.72 | C_29_H_36_O_15_ | [M+Na]^+^ | 647.1946 | -0.62 | 647.1919 | 501.1343, 349.0498, 163.0407 | acteoside | P | 🗸 | 🗸 |  |
| 78^^^ | 11.75 | C_12_H_12_N_2_O_3_ | [M+Na]^+^ | 255.0740 | -6.65 | 255.0723 |  | isomer of 4 | A |  |  | 🗸 |
| 79^^^ | 11.94 | C_31_H_40_O_15_ | [M+Na]^+^ | 675.2259 | -3.40 | 675.2236 | 543.1822, 441.1365, 317.0830, 185.0415 | martynoside | P | 🗸 | 🗸 |  |
| 80^^^ | 11.97 | C_11_H_16_O_3_ | [M+H]^+^ | 197.1172 | -4.93 | 197.1162 | 179.1055, 135.1155, 93.0695 | isomer of 43 | S | 🗸 |  | 🗸 |
| 81 | 12.14 | C_20_H_22_O_8_ | [M+Na]^+^ | 413.1207 | -3.08 | 413.1194 | 398.1229, 380.1458, 163.0405 | isomer of 72 | P | 🗸 | 🗸 |  |
| 82^^^ | 12.28 | C_18_H_21_NO_8_ | [M+NH_4_]^+^ | 397.1605 | 0.24 | 397.1606 |  | strobilanthoside C | A |  |  | 🗸 |
| 83*^^^ | 12.31 | C_9_H_7_NO | [M+H]^+^ | 146.0600 | -4.38 | 146.0594 | 119.0637, 93.0680, 68.0609 | 1*H*-indole-3-carbaldehyde | A | 🗸 | 🗸 | 🗸 |
| 84 | 12.36 | C_16_H_10_N_2_O_2_ | [M+H]^+^ | 263.0815 | -5.80 | 263.0800 |  | isomer of 138/144 | A |  |  | 🗸 |
| 85 | 12.40 | C_29_H_36_O_15_ | [M+Na]^+^ | 647.1946 | -1.99 | 647.1933 |  | isoacteoside | P | 🗸 | 🗸 |  |
| 86^^^ | 12.57 | C_20_H_22_O_8_ | [M+Na]^+^ | 413.1207 | -1.29 | 413.1202 |  | isomer of 72 | P |  |  | 🗸 |
| 87 | 12.58 | C_29_H_36_O_15_ | [M+Na]^+^ | 647.1946 | -2.30 | 647.1931 | 502.1410, 468.1700 | isomer of 77 | P | 🗸 | 🗸 |  |
| 88 | 12.65 | C_17_H_17_NO_9_ | [M+NH_4_]^+^ | 397.1242 | -4.24 | 397.1225 | 262.0703, 148.0378, 136.0611 | isomer of 69 | A | 🗸 | 🗸 |  |
| 89^#^ | 12.70 | C_12_H_12_N_2_O_3_ | [M+H]^+^ | 233.0921 | -2.09 | 233.0916 | 237.0658, 146.0240, 120.0460, 90.0333 | isomer of 4 | A | 🗸 | 🗸 |  |
| 90 | 12.72 | C_17_H_20_N_2_O_6_S | [M+Na]^+^ | 403.0934 | 2.86 | 403.0946 |  | isatindigotindoloside C | A |  |  | 🗸 |
| 91^^^ | 12.82 | C_20_H_22_O_8_ | [M+Na]^+^ | 413.1207 | 0.80 | 413.1210 |  | isomer of 72 | P |  |  | 🗸 |
| 92^#^ | 12.88 | C_17_H_17_NO_9_ | [M+NH_4_]^+^ | 397.1242 | -0.62 | 397.1240 | 262.0703, 148.0378, 136.0611 | isomer of 69 | A | 🗸 | 🗸 |  |
| 93^#^ | 12.93 | C_17_H_17_NO_9_ | [M+NH_4_]^+^ | 397.1242 | 0.99 | 397.1246 | 351.1490, 339.1407 | isomer of 69 | A | 🗸 |  |  |
| 94^^^ | 13.05 | C_11_H_10_N_2_O_2_ | [M+H]^+^ | 203.0815 | -4.86 | 203.0805 |  | isomer of 37 | A |  |  | 🗸 |
| 95 | 13.18 | C_17_H_17_NO_9_ | [M+NH_4_]^+^ | 397.1242 | 3.18 | 397.1255 | 308.1295 | isomer of 69 | A | 🗸 |  |  |
| 96 | 13.22 | C_16_H_18_N_2_O_5_S | [M+Na]^+^ | 373.0829 | -6.24 | 373.0806 |  | isomer of 73 | A |  |  | 🗸 |
| 97 | 13.32 | C_17_H_20_N_2_O_6_S | [M+Na]^+^ | 403.0934 |  | 403.0934 |  | isomer of 90 | A |  |  | 🗸 |
| 98 | 13.36 | C_18_H_14_N_2_O_2_ | [M+H]^+^ | 291.1128 | -6.80 | 291.1108 |  | *N*,*N'*-dimethylindigo | O |  | 🗸 | 🗸 |
| 99^#^ | 13.45 | C_26_H_26_O_11_ | [M+NH_4_]^+^ | 532.1813 | -2.43 | 532.1800 | 514.1679, 502.1690, 378.7631, 353.0076, 311.0903 | procumbenoside L | L | 🗸 | 🗸 |  |
| 100 | 13.48 | C_22_H_22_O_11_ | [M+H]^+^ | 463.1235 | -2.57 | 463.1223 |  | hispiduloside | F |  | 🗸 |  |
| 101 | 13.65 | C_10_H_10_N_2_O_2_ | [M+H]^+^ | 191.0815 | -4.29 | 191.0807 |  | isomer of 122 | A |  |  | 🗸 |
| 102^#^^ | 13.66 | C_15_H_10_N_2_O_3_ | [M+H]^+^ | 267.0764 | -4.88 | 267.0751 | 249.0658, 221.0693, 120.0446, 65.0383 | 3-(2-carboxyphenyl)-4(3*H*)-quinazolinone | A | 🗸 | 🗸 | 🗸 |
| 103 | 13.88 | C_16_H_10_N_2_O_2_ | [M+H]^+^ | 263.0815 | 1.76 | 263.0820 | 245.0723, 227.1076, 146.1011, 71.0845 | isomer of 138/144 | A | 🗸 |  |  |
| 104 | 14.31 | C_17_H_20_N_2_O_6_S | [M+Na]^+^ | 403.0934 | -1.93 | 403.0926 |  | isomer of 90 | A |  |  | 🗸 |
| 105^#^ | 14.39 | C_12_H_12_N_2_O_3_ | [M+Na]^+^ | 255.0740 | 2.06 | 255.0745 | 237.0665, 210.0779 | isomer of 4 | A | 🗸 | 🗸 |  |
| 106^^^ | 14.53 | C_11_H_12_N_2_O_2_ | [M+H]^+^ | 205.0972 | -1.11 | 205.0970 |  | isomer of 8 | A |  |  | 🗸 |
| 107^^^ | 14.55 | C_11_H_12_N_2_O_4_ | [M+Na]^+^ | 259.0689 | -0.15 | 259.0689 |  | 2,3-dihydro-3-hydroxy-4-methoxy-2-oxo-1*H*-indole-3-acetamide | O |  |  | 🗸 |
| 108^^^ | 14.63 | C_11_H_16_O_3_ | [M+Na]^+^ | 219.0992 | -0.32 | 219.0991 |  | isomer of 43 | S |  |  | 🗸 |
| 109^^^ | 14.66 | C_10_H_12_N_2_O_2_ | [M+Na]^+^ | 215.0791 | -4.10 | 215.0782 |  | acanthicifoline | O |  |  | 🗸 |
| 110 | 14.70 | C_10_H_9_NO_2_ | [M+H]^+^ | 176.0706 | -8.54 | 176.0691 |  | isomer of 47 | A |  |  | 🗸 |
| 111^#^ | 14.74 | C_22_H_18_N_4_O_2_ | [M+H]^+^ | 371.1503 | -3.45 | 371.1490 | 309.1439, 207.1222, 161.0416 | 7-[cyano(4-methoxy-1*H*-indol-3-yl)methyl]-3-cyanomethyl-4-methoxy-1*H*-indole | A | 🗸 | 🗸 |  |
| 112^#^ | 14.85 | C_14_H_10_N_2_O_2_ | [M+H]^+^ | 239.0815 | -0.14 | 239.0815 | 211.0866, 132.0448, 120.0445, 108.0437, 92.0497, 65.0385 | 3-(2-Hydroxyphenyl)-4(3*H*)-quinazolinone | A | 🗸 | 🗸 | 🗸 |
| 113^#^ | 14.99 | C_17_H_17_N_3_O_3_ | [M+Na]^+^ | 334.1162 | 1.75 | 334.1168 | 277.1436, 214.9191, 190.9014 | dehydroisolongistrobine | A | 🗸 | 🗸 |  |
| 114^#^ | 14.99 | C_22_H_22_N_2_O_9_ | [M+Na]^+^ | 481.1218 | 1.54 | 481.1225 | 423.6334, 371.7989 | baphicacanthin B | A | 🗸 |  |  |
| 115^^^ | 14.99 | C_16_H_12_N_2_O_4_ | [M+H]^+^ | 297.0870 | -2.16 | 297.0864 |  | 3-Ph, 1-acetoxy-2,4(1*H*,3*H*)-quinazolinedione | A |  | 🗸 |  |
| 116^^^ | 15.19 | C_31_H_40_O_15_ | [M+Na]^+^ | 675.2259 | -1.71 | 675.2247 | 529.1679, 481.1669, 335.1118 | isomer of 79 | P | 🗸 | 🗸 |  |
| 117 | 15.37 | C_16_H_10_N_2_O_3_ | M+H]^+^ | 279.0764 | -2.28 | 279.0758 |  | isomer of 71 | A |  | 🗸 |  |
| 118 | 15.95 | C_31_H_40_O_15_ | [M+Na]^+^ | 675.2259 | -1.71 | 675.2247 | 529.1687, 481.1781, 335.1096 | isomer of 79 | P | 🗸 | 🗸 |  |
| 119^^^ | 16.10 | C_17_H_12_N_2_O | [M+H]^+^ | 261.1022 | -2.66 | 261.1015 |  | 1,2-dihydro-2-(1*H*-indol-3-ylmethylene)-3*H*-indol-3-one | O |  |  | 🗸 |
| 120^^^ | 16.39 | C_17_H_12_N_2_O | [M+H]^+^ | 261.1022 | -3.22 | 261.1014 |  | isomer of 119 | O |  |  | 🗸 |
| 121^#^ | 16.48 | C_13_H_12_N_2_O | [M+Na]^+^ | 235.0842 | 0.85 | 235.0844 | 172.9332, 156.9589,106.9481 | *β*-carboline | A | 🗸 | 🗸 |  |
| 122^#^ | 16.73 | C_10_H_10_N_2_O_2_ | [M+Na]^+^ | 213.0634 | 4.30 | 213.0643 | 185.0706 | 1,2,3,4-tetrahydro-2-oxo-4-quinolinecarboxamide | A | 🗸 | 🗸 |  |
| 123^#^ | 17.03 | C_17_H_14_O_5_ | [M+ NH_4_]^+^ | 316.1179 | -2.61 | 316.1171 | 298.1047,285.0983, 266.0813, 254.0798 | 4'-hydroxy-5,7-dimethoxyflavone | F | 🗸 | 🗸 |  |
| 124 | 17.66 | C_14_H_11_NO_4_ | [M+H]^+^ | 258.0761 | -3.92 | 258.0751 | 159.0892, 93.0727 | baphicacanthin A | A | 🗸 |  |  |
| 125^^^ | 18.04 | C_8_H_11_NO | [M+Na]^+^ | 160.0733 | 4.61 | 160.0740 |  | isomer of 49 | A |  |  | 🗸 |
| 126 | 18.04 | C_18_H_14_N_2_O_2_ | [M+H]^+^ | 291.1128 | -4.48 | 291.1115 |  | isomer of 98 | O |  |  | 🗸 |
| 127 | 18.25 | C_45_H_44_N_2_O_17_ | [M+H]^+^ | 885.2713 | -0.51 | 885.2708 | 739.2145, 415.1279, 163.0369 | strobilanthoside A | A | 🗸 | 🗸 |  |
| 128 | 18.42 | C_33_H_46_O_17_ | [M+Na]^+^ | 737.2627 | -1.26 | 737.2618 | 639.5165, 569.1854, 507.1892, 339.1097, 231.0886, 171.0636 | (+)-lyoniresinol-3*α*-O-*β*-D-apiofura-nosyl-(1→2)-β-D-glucopyranoside | L | 🗸 |  |  |
| 129^^^ | 18.90 | C_16_H_10_N_2_O_3_ | [M+H]^+^ | 279.0764 | -0.45 | 279.0763 |  | isomer of 71 | A |  |  | 🗸 |
| 130 | 18.95 | C_16_H_12_N_2_O_4_ | [M+H]^+^ | 297.0870 | -0.29 | 297.0869 |  | isomer of 115 | A |  |  | 🗸 |
| 131^#^ | 19.33 | C_37_H_46_O_18_ | [M+Na]^+^ | 801.2576 | -0.76 | 801.2570 | 741.2350, 607.191, 571.1790, 377.1211, 193.0462, 151.0746 | isomer of 133 | L | 🗸 | 🗸 |  |
| 132 | 19.46 | C_28_H_34_O_15_ | [M+H]^+^ | 611.1970 | -0.55 | 611.1967 | 551.1786, 177.0540 | isonuomioside A | P | 🗸 | 🗸 |  |
| 133^#^ | 19.46 | C_37_H_46_O_18_ | [M+Na]^+^ | 801.2576 | -0.72 | 801.2570 | 741.2350, 607.191, 571.1790, 377.1211, 193.0462, 151.0746 | 6'-*O*-(1-hydroxy-4-oxocyclohexylacety)acteoside | L | 🗸 | 🗸 |  |
| 134* | 19.54 | C_15_H_8_N_2_O_2_ | [M+H]^+^ | 249.0659 | -4.72 | 249.0647 | 221.0707, 146.0243, 130.0289, 102.0339 | tryptanthrin | A | 🗸 | 🗸 | 🗸 |
| 135^^^ | 19.60 | C_18_H_28_O_4_ | [M+Na]^+^ | 331.1880 | -6.88 | 331.1857 |  | corchorifatty acid B | O |  |  | 🗸 |
| 136^^^ | 19.98 | C_8_H_11_NO | [M+Na]^+^ | 160.0733 | -1.53 | 160.0731 |  | isomer of 49 | A |  |  | 🗸 |
| 137^#^ | 20.51 | C_15_H_14_O_3_ | [M+H]^+^ | 243.1016 | -3.49 | 243.1008 | 228.0807, 201.0563, 187.0410, 166.5679, 159.0438 | rhinacanthone | O | 🗸 |  |  |
| 138* | 20.52 | C_16_H_10_N_2_O_2_ | [M+H]^+^ | 263.0815 | -0.50 | 263.0814 | 235.0845, 219.0906, 190.0639, 132.0434, 77.0381 | indigo | A | 🗸 | 🗸 |  |
| 139 | 20.53 | C_16_H_10_N_2_O_3_ | [M+H]^+^ | 279.0764 | -4.01 | 279.0753 |  | isomer of 71 | A |  | 🗸 |  |
| 140 | 20.53 | C_16_H_12_N_2_O_4_ | [M+H]^+^ | 297.0870 | -3.67 | 297.0859 |  | isomer of 115 | A |  | 🗸 |  |
| 141 | 20.53 | C_8_H_5_NO_2_ | [M+H]^+^ | 148.0393 | -1.83 | 148.0390 | 151.9734, 141.9323, 128.9566, 93.9733, 96.9370, 77.9431, 68.9412, 52.9444 | isomer of 41 | A | 🗸 |  |  |
| 142 | 20.54 | C_8_H_5_NO_2_ | [M+Na]^+^ | 170.0212 | -4.74 | 170.0204 |  | isomer of 41 | A |  | 🗸 |  |
| 143^^^ | 20.58 | C_15_H_10_N_2_O_3_ | [M+H]^+^ | 267.0764 | -0.81 | 267.0762 |  | isomer of 102 | A |  |  | 🗸 |
| 144* | 20.99 | C_16_H_10_N_2_O_2_ | [M+Na]^+^ | 285.0634 | 0.34 | 285.0635 | 245.0731, 144.0437, 120.0439 | indirubin | A | 🗸 | 🗸 | 🗸 |
| 145^#^ | 21.04 | C_12_H_12_N_2_O_3_ | [M+Na]^+^ | 255.0740 | 0.21 | 255.0741 | 237.0659, 209.0703 | isomer of 4 | A | 🗸 | 🗸 |  |
| 146^#^ | 21.04 | C_13_H_12_N_2_O | [M+Na]^+^ | 235.0842 | -3.69 | 235.0833 | 190.0879 | isomer of 121 | A | 🗸 |  |  |
| 147 | 21.27 | C_16_H_10_N_2_O_2_ | [M+H]^+^ | 263.0815 | -1.61 | 263.0811 | 245.0731, 144.0437, 120.0439 | isomer of 138/144 | A | 🗸 |  |  |
| 148 | 24.34 | C_32_H_18_N_4_O_2_ | [M+H]^+^ | 491.1503 | -0.68 | 491.1500 |  | bisindigotin | A |  |  | 🗸 |

A: alkaloids; F: flavonoids; L: lignans; O: others; P: phenylpropanoids; S: sesquiterpene lactones

*Compounds confirmed with standards; ^#^compounds identified in NBLG or NBLJ for the first time; ^^^compounds identified in BBLG for the first time
